# Conserved peptides within the E2 region of Hepatitis C virus induce humoral and cellular responses in goats

**DOI:** 10.1186/1743-422X-6-66

**Published:** 2009-05-27

**Authors:** Mostafa K El-Awady, Ashraf A Tabll, Yasmine S El-Abd, Hassan Yousif, Mohsen Hegab, Mohamed Reda, Reem El Shenawy, Rehab I Moustafa, Nabila Degheidy, Noha G Bader El Din

**Affiliations:** 1 Department of Biomedical Technology, National Research Center, Giza, Egypt; 2 Parasitology and Animal Diseases Department, National Research Center, Giza, Egypt

## Abstract

The reason(s) why human antibodies raised against hepatitis C virus (HCV) E2 epitopes do not offer protection against multiple viral infections may be related to either genetic variations among viral strains particularly within the hypervariable region-1 (HVR-1), low titers of anti E2 antibodies or interference of non neutralizing antibodies with the function of neutralizing antibodies. This study was designed to assess the immunogenic properties of genetically conserved peptides derived from the C-terminal region of HVR-1 as potential therapeutic and/or prophylactic vaccines against HCV infection. Goats immunized with E2-conserved synthetic peptides termed p36 (a.a 430–446), p37(a.a 517–531) and p38 (a.a 412–419) generated high titers of anti-p36, anti-p37 and anti-P38 antibody responses of which only anti- p37 and anti- p38 were neutralizing to HCV particles in sera from patients infected predominantly with genotype 4a. On the other hand anti-p36 exhibited weak viral neutralization capacity on the same samples. Animals super-immunized with single epitopes generated 2 to 4.5 fold higher titers than similar antibodies produced in chronic HCV patients. Also the studied peptides elicited approximately 3 fold increase in cell proliferation of specific antibody-secreting peripheral blood mononuclear cells (PBMC) from immunized goats. These results indicate that, besides E1 derived peptide p35 (a.a 315–323) described previously by this laboratory, E2 conserved peptides p37 and p38 represent essential components of a candidate peptide vaccine against HCV infection.

## Introduction

Hepatitis C virus (HCV) infection is a global blood borne disease that affects almost 3% of the world's population with a morbidity and mortality rates that are second only to HIV among the emerging infections [1]. The highest estimated prevalence of HCV has been reported in Egypt [2,3] with 11–14% of the population chronically infected with the virus. This high prevalence has been attributed to using the intravenous tartar emetic injections in a series of well intended countrywide schistosomiasis control campaigns that occurred from the 1950s until 1980 [2,3] Only 20% or less of initial HCV infections cause acute viral hepatitis severe enough for the patient to seek medical care, however 60–85% of all infections become persistent [4,5]. Individuals with chronic HCV infection usually remain asymptomatic and undiagnosed for decades before chronic hepatitis sometimes leads to severe fibrosis and cirrhosis, hepatic failure, or hepatocellular carcinoma. [6-10]. These long-term complications, along with the large reservoir of infected people, made HCV one of the leading public-health problems. Continuous improvements in transmission prevention and chemotherapeutic regimens are promising, but on their own are unlikely to control this premium cause of chronic liver disease. The current antiviral regimen, a combination of pegylated interferon α and ribavirin, is curative in about half of treated patients depending on the viral and/or host factors. Additionally, this regimen requires prolonged therapy, sometimes with serious side effects, expensive and only a fraction of those with chronic HCV infections meet the criteria for treatment [11]. Intravenous drug users and certain high-risk groups will continue to have an increased chance of exposure to the virus and are at risk f Manns et al., [11] or new infections [12,13]. HCV transmission is likely to persist in areas with limited access to antiviral drugs and poor needle injection and blood product hygiene. Thus, development of a vaccine capable of preventing chronic HCV infection, if not preventing infection altogether, is essential for the control of HCV disease. Vaccine induced antibodies that interfere with viral entry are the protective correlate of many existing prophylactic vaccines. However, for highly variable RNA viruses such as Human immunodeficiency virus (HIV), the genesis of broadly reactive neutralizing antibody (nAb) responses by vaccination has been very difficult reviewed in Phogat et al., [14]. Indeed, HIV has evolved several mechanisms to evade antibody-mediated neutralization, including the masking of conserved regions by glycan, quaternary protein interactions and the presence of immune-dominant variable elements. Therefore, several investigators have focused on E2 glycoproteins (gps) for developing HCV vaccines including purified recombinant glycoproteins (gps) [15,16], modified viral vectors expressing HCV gps [17,18], recombinant virus like particles encoding HCV gp epitopes, and DNA constructs encoding HCV gps [19]. These studies reported that anti-gp responses can be elicited (reviewed in Lechmann and Liang) [20]. However, they did not report on the neutralizing activity of the induced antibodies, but rather several of these reports assessed whether anti-gp responses inhibited the binding of recombinant E2 to cells [15,19,21]. On the other hand, several observations support the hypothesis that neutralizing antibodies (nAb) may help control HCV replication. These included (i) immunization of chimpanzees to elicit gp specific Ab responses induced sterilizing immunity against challenge with homologous virus [22,23]. (ii) recombinant gps induce a response that modulates infection and reduces the rate of progression to chronic disease in chimpanzees [24,25]. (iii) HCV infected patients with antibody deficiencies have accelerated rates of disease progression [26,27]. (iv) passive administration of hyperimmune sera containing Abs capable of neutralizing HCVpp reduced HCV viraemia post-liver transplant [28] and modulated chimpanzee progression rate to chronic disease [29]. Several studies used synthetic peptides derived from various regions of HCV proteins as vaccine candidates proposing that the elicited antibodies would interfere with the viral life cycle [30,31]. In the present study we hypothesize that the sequence motifs located at the amino-terminal region of HVR-1 contains several genetically conserved sequences which may include conformation dependent epitope. The development of antibodies to these motifs may interfere with the mechanisms involved in viral adherence to cell surface or even to viral assembly. We designed and synthesized conserved peptides from this domain used them to immunize goats and purified the goat antibodies for examining their immunogenic and neutralizing properties as candidates for further assessment of HCV peptide vaccine.

## Materials and methods

### Design of the E2 conserved peptides

**Figure 1:**
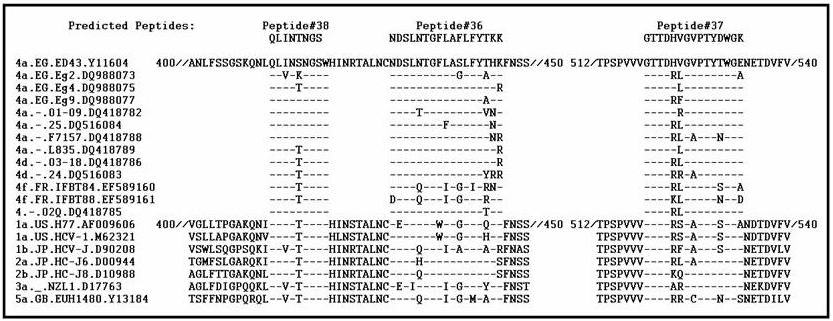
**Alignment of amino acid sequences of the E2 sub-genomic region among various HCV genotypes with special emphasis on subtype (4a)**. Predicted Peptides #38, #36 & #37 in this study are shown on the top of the aligned sequences. A hyphen indicates an amino acid residue identical to that of the HCV genotype 4a.EG.ED43.Y11604 sequence

Three synthetic peptides from the region located C-terminal to HVR-1 of the E2 protein were designed and synthesized. This was done commercially by ANASPEC, Inc, (San Jose CA, USA), in the amide form, using standard solid phase synthesis involving 9-flurenylmethoxy carbonyl chemistry and purified using HPLC as described in our previous study [32]. Amino acid sequences of the E2 region among different HCV genotypes/subtypes were retrieved from the Los Almos hepatitis C sequence database . Three candidate peptides were selected after alignment using Clustal W multiple sequence alignment program at . (Figure 1). Peptides were selected on the basis of sequence conservation among E2 sequences recorded on the HCV data base as in table 1.

**Table 1:** Sequence location results of the predicted peptides.

Peptide	Epitope mapping^1^	Epitope mapping^2^	AA sequence	No. of AA residues
38	29–36	412–419	QLINTNGS	8 mer

36	47–63	430–446	NDSLNTGFLAFLFYTKK	17 mer

37	134–148	517–531	GTTDHVGVPTYDWGK	15 mer

1^st ^column indicates Peptide designation.

1: Represents AA position relative to protein start in H77.

2: Represents AA position relative to polyprotein start in H77.

### Detection of anti E2-peptide immunoglobulin in Chronic HCV patients

A hundred serum samples from chronic HCV patients and 25 samples from healthy individuals who tested negative for anti-HCV antibodies and did not have history of liver disease were used to test the reactivity of the synthetic peptides. Enzyme linked immunosorbent assay (ELISA) was established in house. Briefly, polystyrene micro titer ELISA plates were coated with 50 μl/well of (5 ng/ml) of synthetic peptides p36, p37 and p38 diluted in carbonate/bicarbonate buffer (pH 9.6). The plates were incubated overnight at room temperature and washed three times using 0.05% (v/v) PBS-T20 (pH 7.2). Free active sites were blocked using 0.2% (w/v) nonfat milk in carbonate/bicarbonate buffer. After washing, 50 μl/well from each test sample that was pre-diluted 1:2,000 in PBS were added, and incubated at 37°C for 2 h. After washing,, 50 μl/well of anti-human IgG peroxidase conjugate, diluted in 0.2% (w/v) nonfat milk in PBS-T2 were added and the mixture was incubated at 37°C for 1 h. The amount of coupled conjugate was determined by incubation with 50 μl/well O-Phenylene Diamine (OPD, 0.01%) substrate (Sigma, USA) for 30 min at 37°C. Finally, the reaction was stopped using 3 M HCl and the absorbance was read at 450 nm.

### Production of Caprine polyclonal-mono-specific antibodies

Six Goats were immunized with the synthetic peptides p36, p37 and p38. Each peptide was injected as conjugated to KLH to a pair of goats, 2 goats were injected with 2 ml saline solution at the time intervals of immunization protocol to serve as controls. Each goat was immunized with a unified dose containing 1.5 mg/ml. Equal volumes of diluted KLH – peptide and Freund's complete adjuvant were emulsified and injected subcutaneously into the goat in three different sites. On day 15 and 28, each goat was immunized again with the same protein emulsified with Incomplete Freund's adjuvant. On day 32, bleeding of the goats was done to quantify the titer of relevant immunoglobulin using ELISA. IgG purification was carried out in two steps according procedures of McKinney and Parkinson [33]. To summarize, the first step involves precipitation of albumin and other non IgG proteins with Caprilyic acid (octanoic acid). While the second step involves precipitation of IgG fraction was using ammonium sulphate cut.

### Efficacy of Caprine antibodies to recognize relevant epitopes on HCV particles using Immune-Capture-RT-nested PCR neutralization assay

Thermo well^® ^GOLD PCR tubes (Corning Costar Inc., USA) were coated with serial dilutions of purified Caprine anti-HCV mono-specific IgG. Following a washing step, using 0.05% (v/v) PBS-T20 (pH 7.2), and non specific binding sites was blocked by incubation with 0.2% Bovine serum albumin in PBS at 37°C for 2 hours. Washing by PBS-T20 (pH 7.2) was repeated after the blocking step. Antibodies-coated tubes were incubated with HCV positive serum for 1 hour at 37°C. Serum was aspirated into a 1.5 ml tubes and PCR tubes were washed 3 times and the wash-out was collected into clean collection tubes. Immune-capture RT-PCR was carried out both In-situ and after extraction of viral RNA from the collected fraction. PCR products were electrophoresis on ethidium bromide-stained 1.5% agarose gel. Assessing the specificity of viral binding to anti E2 goat IgG was done through the use of anti-HBV IgG for cross-reaction with HCV particles.

### Stimulation of goat PBMC proliferation with E2-peptides

Five ml blood from immunized (2 animals per each peptide) and 2 non immunized goats (control) were collected on heparinized tubes and PBMC were separated from whole blood using Ficoll separating solution [34]. Cells were washed with PBS and centrifuged at 1600 rpm for 15 min three times. The washed cell pellets were spun down and re-suspended in 1 ml RPMI-1640, supplemented with 10% FCS. Cells were counted and adjusted with RPMI 1640 to be 0.75 million cells/ml media. The cells were plated onto a 24 well plate at 0.5 million cells per well. Cells were incubated with 0, 5, 10, 25, and 50 μg/ml of p36, p37 and p38. The same peptide concentrations were incubated with PBMCs from normal non-immunized goats as negative controls. Phytoheamaglutinine (PHA) was added to culture medium at 5 μg/ml, as positive control for cell stimulation. Cells were cultured in a humidified atmosphere at 37°C, 5% CO2 for 7 days and media were changed every 48 hours.

### FACS analysis

Cells were washed, permeabilized with 0.1% triton X-100 solution (v/v) for 6 min at 4°C and stained with 50 μg/ml propidium Iodide (PI) as a DNA-specific fluorochrome for 30 min at 4°C in a dark place. Cell cycle analysis and cell proliferation (S+ G_2_M) were performed on FACS Caliber flow cytometer.

### Statistical analysis

All statistical analyses were performed using the SPSS 9.0 statistical software program. The statistical significance of difference was considered when p ≤ 0.05.

## Results

### Detection of reactive human IgGs towards the conserved E2 peptides in chronic HCV patients

**Figure 2:**
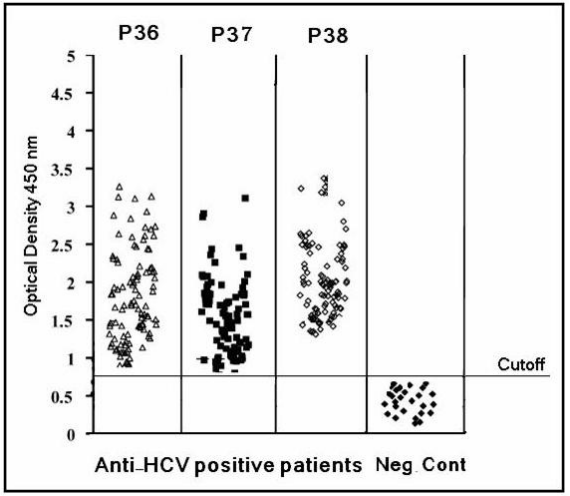
**Reactivity of human IgGs towards the conserved E2-peptides in chronic HCV genotype 4a patients**. The corresponding titers of human IgGs against each tested peptide were determined in 100 HCV patients and 25 healthy control subjects. Levels of antibodies as detected with specific ELISA are depicted as scatter diagram. Cutoff value was calculated from the levels obtained from healthy controls (mean of negative values + 3×S.D).

To answer the question whether the selected conserved E2-peptides were able to recognize specific immunoglobulins in chronic HCV patients, 100 chronic patients and 25 healthy controls were recruited for analysis of specific IgG titers. Using a cutoff of recognition calculated for each peptide (mean of the values obtained with HCV negative sera + 3 × S.D), positive responses were obtained in 100 out of 100 (100%) chronic patients using either of the three peptides p36, p37 and p38. On the other hand neither of the healthy controls displayed positive reactivity towards any of the conserved peptides tested (Figure 2). These results indicate that the selected epitopes were able to induce humoral immune responses during the infection in all the studied patients with genotype 4a.

### Goat IgG levels against multiple doses of HCV E2 peptide epitopes

**Figure 3:**
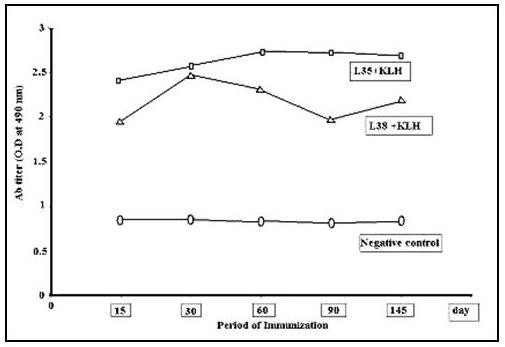
**Goat IgG levels against multiple doses of linear peptide p38 conjugated with KLH**. (P35-KLH and saline treated goats served as positive and negative controls respectively.) Antibody titers were followed for a total of 145 days. The results shown represent the mean values of two goats at each time interval.

To check the sustenance of antibody levels in 2 goats receiving multiple doses of E2 specific peptide, goats were immunized subcutaneously with p38-KLH at days 0, 14 and 28 A pair of goats received p35-KLH (E1 specific peptide that was previously shown to be highly immunogenic and neutralizing, El Awady et al [35] following the same protocol as p38-KLH for comparison. Two goats receiving saline were included as controls. Detectable levels of specific antibodies appeared at the first determination 15 days post immunization, peaked after 30 days and achieved plateau for the next 4 months of the study (i.e. 96 days after the last injection, Figure 3).

### Comparison between titers of anti E2-peptide antibodies in chronic HCV patients and super-immunized goats

**Figure 4:**
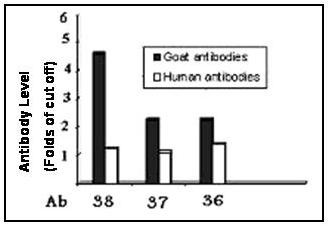
**Comparison between titers of anti E2-peptide antibodies in chronic HCV patients and super-immunized goats**. ELISA plates coated with p36, p37 or p38 were used for determining relevant antibody titers in both chronic HCV patients and goats super immunized with p36-KLH, p37-KLH or p38-KLH. Bars represent means of Ab titer from 100 HCV patients and from 2 goats who received subcutaneous injections of 1.5 mg at days 0, 14 and 28. Cut off values were calculated as means of anti p38-KLH in 25 healthy subjects and in 2 saline injected goats.

To check whether immunization with a single E2-epitope induces specific antibody titers higher than those induced during natural HCV infection, antibody titers against p36, p37 and p38 were determined in 100 chronic HCV patients and in super immunized goats (2 animals/peptide). Mean values of anti p36 and anti p37 were > two folds higher in super immune animals than infected subjects, while anti p38 antibody had > 4 fold higher titer in super immune goats than HCV patients (Figure 4).

### Viral neutralization by anti E2 peptide goat antibodies

**Figure 5:**
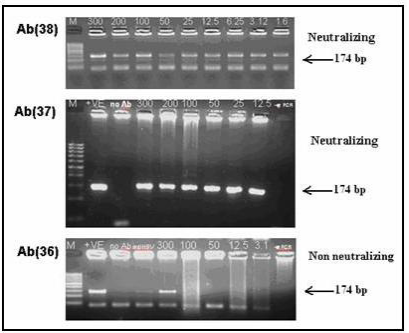
**Viral neutralization by anti E2 peptide goat antibodies**. Purified mono-specific polyclonal antibodies against p36–p38 epitopes were used at 300 to < 12.5 μg/tube to bind HCV from infected sera and the immune-capture activity of each Ab was assessed by RT-nested PCR amplification. The 174 bp amplicon denotes the presence of captured virus. The immune-capture experiment was repeated 3 times with different serum samples. Each Ab displayed the same immune-capture activity with different infected sera. Negative control and binding specificity were assessed by replacing the anti peptide Ab by PBS and anti HBV Ab respectively.

To determine the comparative activities of anti p36, anti p37 and anti p38 in neutralization of HCV, Thermo well^® ^GOLD PCR tubes were coated with serial dilutions (300-1.6 μg/tube) of purified antibodies and allowed to bind the viral particles from patient's sera. After the necessary washing steps the Ab-bound viral particles were determined by RT-nested PCR amplification using HCV specific primers. As shown in figure 5, anti p37 and anti p38 were able to bind HCV at values as low as 12 and 1.6 μg respectively. On the other hand, when anti p36 was used for viral immune-capture it failed to capture the virus at concentrations lower than 300 μg/tube. Tubes coated only with buffer or with anti HBV Ab showed no binding of virus. These results indicate that epitopes p37 and p38 produce specific immunoglobulines in goats with significant viral neutralization capacities, while anti p36 are not neutralizing (Figure 5).

### Effect of E2-peptides on Cell proliferation

**Figure 6:**
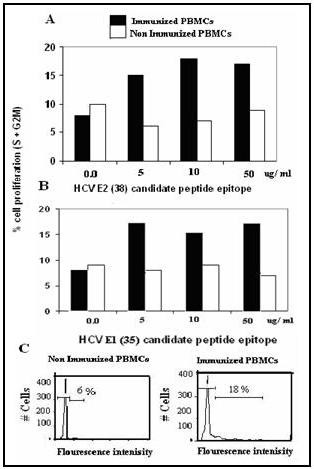
**Effect of p38 epitope on Peripheral blood mononuclear cell proliferation in immunized goats**. Goats were immunized with p38 epitope. PBMC derived from immunized (dark column) and from non immunized (light column) were cultured, stimulated with increasing concentrations (5–50 ug/ml culture) of p38 and analyzed by FACS for cell proliferation (A and C). Results were compared with p35 (B) as a positive control for peptide mediated cell proliferation.

To test whether E2-peptides are able to stimulate cellular response, peripheral blood mononuclear cells (PBMCs) from p38-KLH immunized goats were cultured for 7 days and stimulated with p38 at various concentrations (0–50 μg/ml culture medium), dark boxes). Similar experiments were performed using p35-KLH (E1 peptide that was previously reported by our laboratory to generate neutralizing Abs) for goat immunization and p35 for cell proliferation as positive controls for comparison. PBMC from non immunized goats were cultured and stimulated similar to those cells derived from immunized animals to serve as negative controls (light boxes). Analysis of cell proliferations by flow cytometry showed that cells at (S+G2M) were induced > 2 folds upon stimulation with p38, a proliferative capacity equal to p35 (Figure 6a b, and 6c).

## Discussion

Currently, there is no prophylactic or therapeutic vaccine for HCV; however, there is plenty of evidence supporting the feasibility of such approaches for HCV infection. It is known that natural and protective immunity to HCV exists [36]. The fact that 15–25% of HCV infected individuals are able to spontaneously clear their infection implicates the role of the adaptive arm of immune response in clearance of the virus. Although for reasons that have yet to be understood, convalescent humans are not protected against acute HCV infection. However, the majority of convalescent humans are protected from the progression of infection to chronic state [37]. Since it is the chronic state of HCV infection that is associated with pathogenicity of the virus, this argues for the feasibility of a prophylactic vaccine, able to induce HCV specific immune responses similar to those elicited in convalescent individuals and would be able to protect naïve individuals post infection. Genetic heterogeneity in HCV [38,39], and other RNA viruses such as HIV and Influenza, plays an important role in immune escape and in the establishment of persistent infection. Besides, non neutralizing antibodies were shown to mask the neutralizing antibodies (nAbs) in chronic HCV infection thus explaining low rates of viral clearance. Therefore, it is generally assumed that cross-reactive nAb responses targeting conserved regions of the viral gps would be better able to neutralize the viral quasi-species present within an infected individual. In the present study, we designed and synthesized 3 peptides derived from conserved E2 epitopes on the bases of sequence data available for genotype 4a quasi-species as well as alignments with viral subtypes reported in the HCV database within the NH2-terminal region of the HVR-1 of E2 protein. The current peptides were selected to be genetically conserved at least among viral subtypes infecting the local population, predominantly 4a. The present experimental data confirmed the conservation of selected peptides via their ability to react with corresponding Abs in 100% of the studied local cases of HCV infection. These experiments directed our attention towards the question why these Abs were not able to clear the virus and permitted progression to the chronic state. In support to the hypothesis made by von Hahn et al., [40-42]. We assume that co-existence of non neutralizing Abs (anti p36 in this study) side by side with nAbs (anti p37 + anti p38) may lead to hindrance of neutralizing activity of nAbs. Recent reports of targeting antibody responses to the HCV E2 hyper variable region have elicited low level strain-specific nAb responses [43,44]. These results encouraged us to hypothesize that the low titers of nAbs, perhaps due to exhaustion of humoral response to a multiple epitope vaccination, made them not sufficient for viral neutralization. The results presented herein suggest that hyper-immunization with a specific single E2 epitope elicited higher antibody titers than those generated during chronic viral infection and further deepen our believe that the fewer the number of nAbs used the stronger humoral response and the more chance for viral clearance exists. Elucidation of the neutralization epitopes on the surface of E2 gps is of great interest for the development of an efficient vaccine. Several human anti-E2 antibodies have been reported with cross-reactive neutralizing activity and the majority appears to recognize conformation dependent epitopes [45,46]. This study demonstrates that immunization of goats with synthetic peptides derived from HCV E2 gps can elicit polyclonal antibody responses some of them were capable of neutralizing HCV virions in infected sera. These data further suggest the presence of an immunodominant conserved epitopes within the E2 gp which encompasses motifs from linear epitopes. Since HCV specific T cell responses are required besides humoral responses to assess the efficacy of peptide vaccination, Klade et al., [30] demonstrated that HCV IC41 peptide vaccine induced T-cell responses in HCV difficult to treat patients, where the strongest responses were associated with HCV RNA decline. In the current study, immunization of goats with KLH conjugated peptides induced significant HCV specific cellular response. Although gamma interferon secreting CD+4 and CD+ 8 cells were not analyzed in goats, we have demonstrated ~3 fold increase in HCV antigen specific leucocytes proliferation indicated that our candidate epitope E2 (p38) vaccine was able to induce cellular immune response, which was critical in viral clearance. These data are in agreement with the results of Zhu et al., [47]. The ability of selected peptides to induce strong and specific humoral and cellular immune responses makes them potential candidates for designing a prophylactic and therapeutic vaccine against HCV. Taken together the results of humoral immunity, viral neutralization and specific cellular responses suggest that p37 and p38 together with p35 (E1 derived peptide published earlier, El Awady et al., [32,35] are candidate vaccine components for further studies.

## Competing interests

The authors declare that they have no competing interests.

## Authors' contributions

ME conceived the study, participated in its design and coordination, wrote the final version of the manuscript and supported partial financing. AT participated as a PI of the project supporting the study, wrote the draft of  the  manuscript,  and  followed  up  all  technical  steps.  YE participated  in  designing  the E2  conserved  peptides,  production  of  goat  polyclonal mono-specific  antibodies  and  in  cell  proliferation  assay.  HY  participated  in  immunizing  the  goats  and  in  immunoassays.   MH  carried  out  Immune-Capture-RT nested  PCR.  MR  performed  IgG  purification.  RE and RM carried  out  the immunoassays.  ND  participated  in  animal  selection  and  antigen  immunization.  NB participated in RT-PCR for HCV RNA and ms editing. All authors read and approved the final manuscript
